# Μetal Uptake by Sunflower (*Helianthus annuus*) Irrigated with Water Polluted with Chromium and Nickel

**DOI:** 10.3390/foods6070051

**Published:** 2017-07-17

**Authors:** Vasiliki Stoikou, Vangelis Andrianos, Sotiris Stasinos, Marios G. Kostakis, Sofia Attiti, Nikolaos S. Thomaidis, Ioannis Zabetakis

**Affiliations:** 1 Laboratory of Food Chemistry, Department of Chemistry, University of Athens, 15771 Athens, Greece; vstoikou@chem.uoa.gr (V.S.); vandrianos@chem.uoa.gr (V.A.); sstas@teemail.gr (S.S.); 2 Laboratory of Analytical Chemistry, Department of Chemistry, University of Athens, 15771 Athens, Greece; makostak@chem.uoa.gr (M.G.K.); sofia-attiti@chem.uoa.gr (S.A.); ntho@chem.uoa.gr (N.S.T.); 3 Department of Biological Sciences, University of Limerick, V94 T9PX Limerick, Ireland

**Keywords:** chromium, nickel, sunflower, sunflower oil, metal uptake, water, safety

## Abstract

The water aquifers of the regions of Asopos River in Viotia and Messapia in Evia (Greece) have been contaminated with hexavalent chromium (Cr (VI)) and bivalent nickel (Ni (II)). Given that these areas are the two biggest tuber producing regions of Greece, in our previous work, the cross-contamination of the food chain with these two heavy metals was quantified. In the present study, the potential of sunflower (*Helianthus annuus*) cultivation in these regions is evaluated. The scope of our study was to investigate the uptake of chromium and nickel by sunflower, in a greenhouse experiment. The study included two cultivation periods of plants in six irrigation lines with different levels of Cr (VI) and Ni (II) ranging from 0 μg/L (control) to 10,000 μg/L. In all plant parts, statistically significant increased levels of Cr (VI) and Ni (II) were found when compared to control ones. Also, a positive correlation, both for Cr and Ni, between levels of heavy metals in irrigation water and plants was observed. Following European Food Safety Authority recommendations, the obtained oil was evaluated as safe for consumption, therefore, sunflower cultivation could be a valid bioremediation solution for the Asopos and Messapia regions.

## 1. Introduction

Asopos River in Viotia and Messapia in Evia are the two main tuber (i.e., carrot, potato, and onion) producing regions in Greece. The water aquifer of both areas is polluted with heavy metals due to proximate industrial and mining activities. As a result, the irrigation water used in the food production is contaminated with hexavalent Chromium (Cr (VI)) and bivalent Nickel (Ni (II)). In a greenhouse experiment simulating the irrigation parameters of these regions, it was found that both Cr and Ni (present in irrigation water) can cross-contaminate the food tubers [1]. This cross-contamination also affects the nutritional value of food products [2].

Phytoextraction is a remediation green technology that uses plants to remove, reduce, degrade, or immobilize heavy metals from polluted soil and water. As a remediation technology, it takes advantage of the ability of plants to concentrate elements and compounds from the environment and to metabolize various molecules in their tissues. The success of a phytoextraction process depends on sufficient plant yield (aerial parts) and high metal concentrations in plant parts [3]. Water pollution with heavy metals has been a worldwide concern, as it poses great threats to crop production causing significant cross-contamination of the food chain [4], resulting in negative impacts on human health [5]. As an emerging green technology, phytoextraction has increasingly received widespread attention [6]. The success depends on the growth stage, during which biomass production and accumulation is more intense [7]. The choice of the appropriate method of remediation is governed by the mobility, distribution, and speciation of the toxic metals in soils and plants. 

A variety of anthropogenic factors can be responsible for heavy metal pollution in soils. Heavy metals are very persistent in the environment, they do not biodegrade or thermodegrade, and they accumulate to toxic levels. The cross-contamination of food by Ni and Cr is an emerging nutritional hazard [8]. Although Ni is an essential metal and plays important roles in plant metabolism, Ni toxicity has become of particular concern due to its increased industrial use [9]. More specifically, nickel in high doses and certain forms is toxic and is known to be a human carcinogen, targeting human body systems such as cardiovascular, dermal, immunological, and respiratory. Meanwhile, chromium is of special interest because it is both an essential nutrient and a carcinogen. Chromium is an essential trace element in the human diet. It is poorly absorbed, and the concentrations of chromium in various tissues and fluids are normally quite low. There is no conclusive evidence yet of an essential role of Cr in plant metabolism. Some plant species can accumulate relatively large amounts of the element in their shoots. These are termed as ‘Cr accumulators’ [10].

Sunflower (*Helianthus annuus*) is cultivated as a food and feed crop as well as for bioenergy production, and it has an important role as a plant. It is also being investigated for its ability to remove contaminants from soil. Sunflower can accumulate Pb, Cu, and Cd in the shoots [6]. Sunflower’s shoots and roots exhibited the highest Co and Cr uptake compared to other tested species in any investigated soil. The time required for remediation depends on the type of the metal, the extent of metal pollution, and the length of growing season [11]. Sunflowers were also screened for their capacity to tolerate different concentrations of chromium, hexavalent Cr (VI), and trivalent Cr (III). Sunflower, due to its big biomass, is also recommended for rhyzofiltration and phytoremediation purposes [12]. Studies have reported that the effects of reductants on Cr (VI) phytoremediation clearly influenced Cr concentrations in the roots and shoots of plant *Ipomoea aquatica*. The applications of S_2_O_3_^2−^, Fe^0^, and Fe^2+^ at low doses notably increased root Cr concentrations. However, high reductant concentrations decreased bioaccumulation of Cr in the roots and shoots of the plant [13]. Excess Ni has been reported to cause leaf necrosis and chlorosis of plants. The uptake of Ni by plants depends on Ni^2+^ concentrations, plant metabolism, the acidity of soil or solution, the presence of other metals, and organic matter composition [9]. *Helianthus annuus* which was grown in a soil substrate to which nickel was introduced at 25, 50, 75, and 150 mg/kg, accumulated the highest amounts of this metal. For sunflowers grown on a former uranium mining site, 140–170 days of growth were found to be optimal to reach a maximum extraction for all 25 investigated elements (Cd, Co, Cr, Cu, Fe, Mn, Ni, Pb, Th, U, Zn, and rare earth elements) [14]. 

Given the polluted water aquifers in the tuber producing areas in Greece, this study aimed to evaluate the potential of sunflower, as a Cr and Ni phytoaccumulator, in order to give an alternative farming suggestion. Our working hypothesis was that Cr and Ni, given their hydrophilic character, would not accumulate in lipid fractions of sunflower oil. With our work, reported here, we studied the uptake of Cr and Ni by roots, shoots, leaves, and blossoms of *Helianthus annuus* and assessed the levels of Cr and Ni in the obtained sunflower oil.

## 2. Materials and Methods 

### 2.1. Plant Cultivation in a Greenhouse

Seeds of *Helianthus annuus* were collected from a local agricultural centre, and for the purposes of this study, six irrigation lines (i.e., six 300 L plastic tanks, six pumps, and six series of three tubs per series) were constructed in a greenhouse in the Department of Chemistry at Athens University, Greece. The first cultivation period was from March to July 2015 where the sunflowers were planted in soil and then irrigated by water containing different levels of Cr (VI) and Ni (II), prepared by solid K_2_Cr_2_O_7_ and NiCl_2_∙6H_2_O, respectively. The six lines carried risk elements as follows: 0 μg/L (control), 100 μg/L, 500 μg/L, 1000 μg/L, 5000 μg/L, and 10,000 μg/L in tap water solutions of both Cr (VI) and Ni (II). All the plants were fertilized as described before [1]. The values of the main physicochemical parameters of control (tap) water were: pH = 8.3, turbidity = 7.6 NTU, electrical conductivity (EC) = 295 μS/cm, total dissolved solids (TDS) = 156 mg/L, Na = 4.6 mg/L, K = 0.8 mg/L, Pb = 0.3 μg/ L, Hg = 0 μg/L, Cd = 0 μg/L, Cr = 0 μg/L). The physicochemical properties of the soil used were determined as: pH = 8.2 ± 0.1, conductivity = 2.89 ± 0.33 mS/cm, CaCO_3_ = 9.49 ± 0.58 percent, organic matter = 1.91 ± 0.35 percent, and the grain composition of soil was: sand = 38.6 ± 1.9 percent, clay = 5.9 ± 1.1 percent, and sludge = 55.4 ± 2.3 percent. The soil was characterized as silt-clayey. 

Plants were harvested as a whole and carefully separated into four different parts with a stainless steel cutter. On average, six sunflowers per tub were taken and separated to roots, shoots, leaves, and blossoms, and each part was sliced into very small pieces with a knife. Afterwards, roots, shoots, leaves, and blossoms were separately dried at 40 °C.

A representative quantity of 8–10 g per plant was weighed and put in plastic vessels, refrigerated for 24 h in −80 °C, freeze dried in −55 °C and −1 bar pressure for 48 h, and pulverized by mortar and pestle. Samples of 0.5 g per pulverized plant were diluted in 5 mL of suprapure HNO_3_ (65 percent *v*/*v*), 1 mL of H_2_O_2_, and microwave digested in a three step digestion program (150 °C for 7 min, 170 °C for 10 min, 180 °C for 10 min). After digestion, each sample was diluted to 20 mL final volume by distilled water. Blank samples containing only suprapure nitric acid and hydrogen peroxide and samples containing Standard Reference Material NIST 1573a (tomato leaves) were also digested and analyzed to verify the credibility of the analysis.

The second cultivation period was from March 2016 to July 2016, following the same methodology and irrigation lines. Blossoms were harvested as a whole and carefully separated from seeds. Afterwards, seeds were dried at 40 °C followed by grinding and extraction with hexane in order to extract the oil. Oil samples were diluted in 5 mL suprapure HNO_3_ (65 percent *v*/*v*) and 1 mL of H_2_O_2_ and microwave digested in a three step digestion program as above. After digestion, each sample was diluted to 20 mL final volume by distilled water. Oil market samples were also analyzed. 

For each cultivation period (March–July 2015 and March–July 2016), each tub was totally irrigated with about 750 L of water with Cr (VI) and Ni (II) concentrations varying from 0 to 10,000 μg/L. The total mass of Cr (VI) and Ni (II) added in each tub for the irrigation watering lines of 0, 100, 500, 1000, 5000, and 10,000 μg/L was 0, 75, 375, 750, 3750, and 7500 mg, respectively. During the whole period of the experiment, no symptoms of toxicity were observed in any plant. 

For Cr: average concentration of total Cr was 127.7 ± 2.7 mg/kg before the first cultivation and 129.1 ± 2.6 mg/kg after the first cultivation; 129.1 ± 2.6 mg/kg before the second cultivation and 129.9 ± 2.6 mg/kg after the second cultivation. For Ni: average concentration of Ni was 162.0 ± 5.3 mg/kg before the first cultivation and 162.9 ± 5.2 mg/kg after the first cultivation; 162.9 ± 5.2 mg/kg before the second cultivation and 163.7 ± 5.3 mg/kg after the second cultivation.

Each tub contained 500 kg of soil and the maximum levels of metals added to the soil, i.e., irrigation watering line of 10,000 μg/mL, were 7500 mg or 15 mg/kg. As discussed in our previous work [1], the Cr and Ni quantities added in soil are relatively small in relation to the existing levels of Cr and Ni already present in the soil. In other words, there are already considerable natural “background” levels of both metals in the soil, before irrigation with Cr and Ni polluted water [1]. This is the reason that the soil levels of Cr and Ni have not been increased during the irrigation experiments. It could be thus suggested that irrigated heavy metals were either absorbed by the plants or depleted.

### 2.2. Reagents and Instrumentation

The materials used for Cr and Ni determination were, nitric acid (65 percent *v*/*v*, suprapure grade from Merck, Darmstadt, Germany), hydrogen peroxide (H_2_O_2_), ultrapure water (Millipore, Cork, Ireland, MilliQ), Standard Reference Material NIST 1573a (tomato leaves, from National Institute of Standard and Technology for plants analysis), and commercially available sunflower oil samples. The instrument used for elemental determination in roots, shoots leaves, blossoms, and oils was a Perkin Elmer SIMAA6000 simultaneous multi-elemental atomic absorption spectrometer with THGA graphite furnace and a Zeeman background corrector. The digestions were performed in a microwave oven MARS X Press. The certified mass fractions by National Institute of Standard and Technology (NIST) Of Standard Reference Material 1573a (tomato leaves), for Cr and Ni were estimated 1.99 ± 0.06 and 1.59 ± 0.07 μg/g, respectively. The Cr and Ni concentrations of Standard Reference Material for plants NIST1573A (tomato leaves) in our experiment were measured as 1.65 ± 0.37 μg/g and 1.39 ± 0.72 μg/g plant dry matter (*n* = 13) indicating that the results were reliable.

### 2.3. Gas Chromatographic Analysis of Fatty Acids in Sunflower Oil

#### 2.3.1. Reagents and Instruments

All reagents and solvents were of analytical grade purchased from Merck (Darmstadt, Germany). Fatty acid methyl ester standards were of Gas Chromatography (GC)-quality and supplied by Sigma-Aldrich (St. Louis, MO, USA).

#### 2.3.2. Gas Chromatographic Analysis

Fatty acid methyl esters (FAME) of 35 mg of all oil samples were prepared using a solution of 0.5 N KOH in CH_3_OH (KOH-CH_3_OH method, reaction time 5 min) and extracted with *n*-hexane. The fatty acid analysis was carried out using the internal standard method [15]. A 5-point calibration curve was prepared using five solutions of heptadecanoic (17:0) acid methyl ester and heneicosanoic (21:0) acid methyl ester in ratios of 500:1000 (*v*/*v*), 500:500 (*v*/*v*), 500:200 (*v*/*v*), 500:100 (*v*/*v*), and 500:50 (*v*/*v*), respectively. Five injections of 1 μL of each solution were analyzed with a Shimadzu CLASS-VP (GC-17A, Kyoto, Japan) gas chromatograph equipped with a split/splitless injector and flame ionization detector. The ratio of the mean area (21:0) to that of the internal standard (17:0) was used as the *y*-axis variable of the calibration curve, while the concentration (mg kg^−1^) of 21:0 was used as the *x*-axis variable of the calibration curve. The equation that described the calibration curve was: *y* = 0.0012*x* + 0.0210, with *r* = 0.996. The ratio of the area of the analyte peak to that of the internal standard represents the y value at the above equation, and subsequently the *x* value represents the analyte concentration of the fatty acid in the unknown mixture. Separation of fatty acid methyl esters was achieved on an Agilent J&W DB-23 fused silica capillary column (60 m × 0.251 mm i.d., 0.25 μm; Agilent, Santa Clara, CA, USA). The oven temperature program was: initially 120 °C for 5 min, raised to 180 °C at 10 °C min^−1^, then to 220 °C at 20 °C min^−1^, and finally isothermal at 220 °C for 30 min. The injector and detector temperatures were maintained at 220 and 225 °C, respectively. The carrier gas was high purity helium with a linear flow rate of 1 mL min^−1^ and split ratio 1:50. Fatty acid methyl esters were identified using FAME standards as described previously [15].

### 2.4. Statistical Methodology

The results were statistically processed with IBM SPSS 23 (SPSS Inc., Chicago, IL, USA) and the figures were produced using STATACORP Stata 13 (StataCorp., College Station, TX, USA). The distributions of values diverged from the normal distribution, as shown by the relevant graphic control PP plot in SPSS 23. The levels of Cr and Ni in plants have been calculated as the median values. The statistical evaluation of the differences of Cr and Ni levels between the water concentrations of 0 μg/L and 100 μg/L, 500 μg/L, 1000 μg/L, 5000 μg/L, and 10,000 μg/L was based on the Mann-Whitney non-parametric control. The same non-parametric test was implemented for the “two by two” comparisons between the concentrations of the two heavy metals (Cr, Ni) in irrigation water, while Bonferroni and Sidak corrections were used for the correction of the influence of multiple comparisons (increase of “type I” error) [16]. The non-parametric rank correlation coefficient of Spearman (rho) was also used for the investigation of the relationship between the levels of Cr and Ni in different plants, separately for every metal’s concentration in irrigation water. In order to approximate the association between Cr and Ni within each plant, as well as between each of these heavy metals and the corresponding concentration in the irrigation water, locally weighted scatter plot smoothing (Lowess) was used. The limit of statistical significance was 0.05. When | rho |> 0.3 and *p*-value < 0.05, then the relationship is statistically significant. One-way analysis of variance (ANOVA) using the Office Excel 2010, was used in order to find the statistically significant differences between the fatty acids in oil samples in different irrigation water lines. Differences were considered to be statistically significant when the *p*-value was lower than 0.05. 

## 3. Results

### 3.1. Levels of Cr and Ni in Different Plant Parts

In shoots, the highest Cr absorption was observed in the 10,000 μg/L irrigation line (6.011 μg/g dry matter). In blossoms, the maximum Cr absorption was measured in the 500 μg/L and the 10,000 μg/L irrigation lines (3.142 and 3.087 μg/g dry matter, respectively). Chromium in leaves in the 10,000 μg/L irrigation line was measured at 16.78 μg/g dry matter and it was 2.79 times higher than Cr absorbance in shoots. It should also be noted that Cr in sunflower leaves has been measured in amounts greater than 2 μg/g [17]. While chromium toxicity studies in plants (especially spinach) suggest that the leaves have reduced growth, browning, and chlorosis [18], in our experiment, the sunflower leaves showed very limited browning. For roots, the maximum absorption of Cr was observed in the 5000 μg/L irrigation line (and not in the 10,000 μg/L irrigation line) (14.76 × 10^2^ μg/g dry matter) (Supplementary Materials Figure S1). This could be explained on the basis that the highest levels of heavy metals in water may have caused minor phytotoxic effects to the plants that reduced the absorption of heavy metals. These results are in agreement with our previous work on food tubers [1]. The largest amount of chromium appears to accumulate in sunflower roots. Despite this fact, the roots showed no toxicity, chlorosis, or necrosis after exposure to chromium and nickel, compared to the roots of control plants (0 μg/L). The Ni levels in shoots, leaves, and roots were higher in tubs obtained from the 10,000 μg/L irrigation line, measured as 10.73 μg/g dry matter, 11.01 μg/g dry matter, and 127.7 μg/g dry matter, respectively). Blossoms showed highest nickel absorption in the 5000 μg/L irrigation line, measuring at 8.606 μg/g dry matter, suggesting that the highest levels of heavy metals in water may have been phytotoxic for the sunflower plants [1].

### 3.2. “Two by Two” Comparisons

The statistical significances (*p*-values) of the differences in the levels of chromium and nickel in the “two by two” comparisons between 0 μg/L and 100 μg/L, 500 μg/L, 1000 μg/L, 5000 μg/L, and 10,000 μg/L have been measured; Bonferroni and Sidak corrections were used for the correction of the influence of multiple comparisons and the corrected levels of statistical significance are given in Supplementary Table S1 (* level of significance *p* = 0.01 (Bonferroni correction), # level of significance *p* = 0.102062 (Sidak correction)).

For roots, statistically significant differences in the Cr levels were observed between concentrations of 0 μg/L and 100 μg/L, 500 μg/L, 1000 μg/L, and 5000 μg/L; while no statistically significant differences in levels of Ni were observed in concentrations between 0 μg/L and 500 μg/L or 1000 μg/L. Statistically indicative differences in Ni levels were observed in the roots between 0 μg/L and 5000 μg/L. Statistically significant differences in the Cr levels were observed between concentrations of 100 μg/L and 500 μg/L, 1000 μg/L, and 5000 μg/L; while no statistically significant differences in Ni levels were found between concentrations of 100 μg/L and 1000 μg/L. Statistically indicative differences in Ni levels were observed in concentrations between 100 μg/L and 5000 μg/L. Statistically significant differences in the Cr levels were also noticed between concentrations of 500 μg/L and 1000 μg/L and 5000 μg/L; while no statistically significant differences in Ni levels were found between concentrations of 500 μg/L and 5000 μg/L. Statistically significant differences in the Cr levels were observed between concentrations of 1000 μg/L and 5000 μg/L, while for Ni between 1000 μg/L and 10,000 μg/L, no significant differences were noted. Statistically indicative differences in Cr levels were noticed between concentrations of 5000 μg/L and 10,000 μg/L. 

For shoots, statistically significant differences in the Cr levels were observed between concentrations of 0 μg/L and 500 μg/L, 1000 μg/L, 5000 μg/L, and 10,000 μg/L; while no statistically significant differences in levels of Ni were observed between concentrations of 0 μg/L and 100 μg/L. Statistically indicative differences in Ni levels were observed in the shoots between the concentrations 0 μg/L and 1000 μg/L and 5000 μg/L. Statistically significant differences in the Cr levels were noticed between concentrations of 100 μg/L and 500 μg/L, 1000 μg/L, 5000 μg/L, and 10,000 μg/L. Statistically indicative differences in Ni levels were observed between concentrations of 100 μg/L and 10,000 μg/L. Statistically significant differences in the Cr levels were found between concentrations of 500 μg/L and 5000 μg/L and 10,000 μg/L; while the Ni levels were statistically significantly different between 500 μg/L and 10,000 μg/L. Statistically indicative differences in Ni levels were observed between concentrations of 500 μg/L and 5000 μg/L. Statistically significant differences in the Cr and Ni levels were noticed between concentrations of 1000 μg/L and 10,000 μg/L. Statistically indicative differences in Ni levels were observed in the shoots between concentrations of 1000 μg/L and 5000 μg/L. Statistically significant differences in the levels of Cr were shown between concentrations of 5000 μg/L and 10,000 μg/L. 

For leaves, statistically significant differences in the Cr levels were observed between the concentrations of 0 μg/L and 100 μg/L, 1000 μg/L, and 5000 μg/L; whereas no statistically significant differences in levels of Ni were noted between 0 μg/L and 500 μg/L, 1000 μg/L, and 5000 μg/L. Statistically indicative differences in Cr levels were observed between the concentrations of 0 μg/L and 500 μg/L and 10,000 μg/L. Statistically significant differences in the Cr levels were observed between concentrations of 100 μg/L and 500 μg/L, 1000 μg/L, 5000 μg/L, and 10,000 μg/L; while statistically significant differences in Ni levels were found between concentrations of 100 μg/L and 5000 μg/L and 10,000 μg/L. Statistically significant differences in the Cr levels were observed between concentrations of 500 μg/L and 1000 μg/L and 5000 μg/L, while for Ni, such differences were observed between 500 μg/L and 10,000 μg/L. Statistically indicative differences in Cr levels were observed between concentrations of 500 μg/L and 10,000 μg/L. Statistically significant differences in the Cr levels were also observed between concentrations of 1000 μg/L and 5000 μg/L, while for Ni between 1000 μg/L and 10,000 μg/L, no significant differences were noted. Statistically significant differences in Ni levels were noticed between concentrations of 5000 μg/L and 10,000 μg/L, whereas statistically indicative differences in Cr levels were found between concentrations of 5000 μg/L and 10,000 μg/L.

For blossoms, no statistically significant differences in levels of Cr were observed between 0 μg/L and all irrigation water lines. Statistically indicative differences in Ni levels were observed between 0 μg/L and 1000 μg/L and 5000 μg/L. Statistically indicative differences in Cr and Ni levels were observed between 100 μg/L and 10,000 μg/L; while statistically significant differences in the Ni levels were noticed between concentrations of 100 μg/L and 1000 μg/L and 5000 μg/L. Statistically significant differences in the Ni levels were observed between concentrations of 500 μg/L and 1000 μg/L and 5000 μg/L. Statistically significant differences in the Cr and Ni levels were also observed between concentrations of 1000 μg/L and 5000 μg/L and 10,000 μg/L.

### 3.3. Spearman’s Correlation

**Figure 1:**
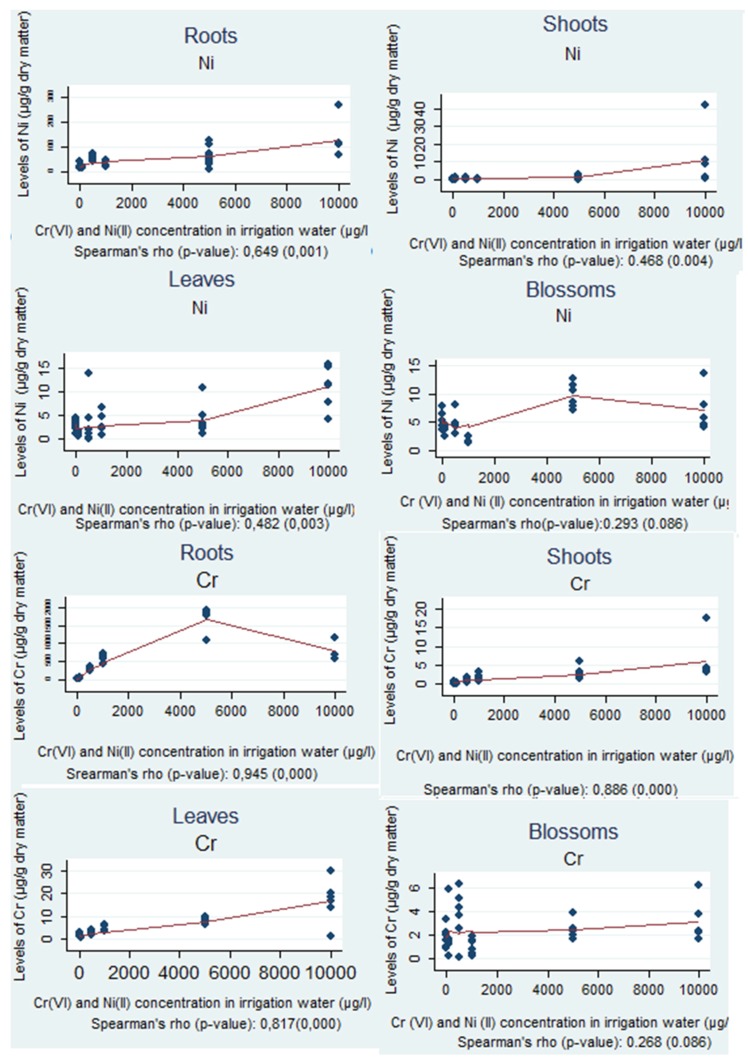
Correlations between the levels of chromium and nickel within each part of the plant, for every irrigation water concentration.

The correlations between the levels of Cr and Ni within each part of the plant, for every irrigation water concentration, separately, are shown in Figure 1.

In Table 1, a positive correlation, characterized as statistical significance, between the levels of Cr and Ni in irrigation water and in roots, shoots and leaves is shown; the *p*-value was below the significance level and the non-parametric correlation coefficient was above 0.3 (Spearman’s rho > 0.3).

In the sunflower roots, according to the statistical analysis, the correlation between the levels of Cr was very strong with Spearman’s rho equal to 0.945 and the significance level (*p*-value) equal to zero. In contrast to the above results, the correlation between Ni levels can be simply characterized as strong, since the non-parametric correlation coefficient, Spearman's rho, was 0.649 and the significance level (*p*-value), was less than 0.05.

In the shoots, Cr levels had a very strong correlation, since the non-parametric correlation coefficient, Spearman's rho, was 0.886 and the significance level (*p*-value), was less than 0.05. For Ni levels, the correlation can be characterized as moderate, because the non-parametric correlation coefficient (Spearman’s rho) was 0.468 with a significance level (*p*-value) of 0.004. 

In the leaves, the correlation between the Cr levels of the plant was also very strong with the non-parametric correlation coefficient (Spearman’s rho) 0.817 and the significance level (*p*-value) below 0.05. For Ni, the correlation can be categorized as moderate with Spearman’s rho at 0.482 and *p*-value equal to 0.003. 

Finally, in the blossoms, the correlation between the levels of Cr was weak with Spearman’s rho equal to 0.268 and the significance level (*p*-value) was 0.086, whereas for Ni, the correlation can be simply characterized as weak, since the non-parametric correlation coefficient, Spearman's rho, was 0.293 and the significance level (*p*-value) was 0.057.

The correlations between Cr and Ni concentrations in roots, shoots, leaves, and blossoms, for all irrigation lines, are given in Table 2.

Statistically significant positive and very strong correlations (Spearman’s rho = 0.986) were observed between the levels of Cr and Ni in shoots (*p*-value = 0.000) irrigated with a water concentration of 10,000 μg/L, in leaves (Spearman’s rho = 0.943) irrigated with water concentration 0 μg/L (*p*-value = 0.005), and in blossoms (Spearman’s rho = 0.829) irrigated with water concentration of 10,000 μg/L (*p*-value = 0.042). All the other correlations, as shown in Table 2, between the levels of chromium and nickel, can be characterized as positive or negative and not statistically significant since the level of statistical significance (*p*-value) was over the limit of 0.05. In roots and for the 500 μg/L irrigation line, there was no correlation at all between the levels of Cr and Ni.

### 3.4. Levels of Cr and Ni in Sunflower Oils

The levels of Cr and Ni in the sunflower oils derived from the different irrigation water lines and a commercial available sample are presented in Table 3. Three samples of sunflower oils were analyzed for each irrigation line using code names such as 0.1, 0.2, and 0.3 for the control irrigation line; 1.1, 1.2, and 1.3 for the 100 μg/L irrigation line, etc. 

In all samples, the levels of Ni were found to be below both the LOD (Limit of detection) and LOQ (limit of quantification) values (LOD = 0.078 μg/g and LOQ = 0.234 μg/g for nickel). The Cr levels could be quantified in all samples of the control irrigation line and for the 100, 1000, 5000, and 10,000 μg/L irrigation lines. In all samples of the 500 μg/L irrigation line, the levels of Cr were below LOQ (LOD = 0.008 μg/g, LOQ = 0.024 μg/g of sample for chromium).

### 3.5. Levels of Fatty Acids in Sunflower Oils

The fatty acid profiles of sunflower oils are presented in the Table 4. As described before (Section 3.3), three samples of sunflower oils were analyzed for each irrigation line.

Considerable amounts of palmitic acid (16:0, R_t_ = 18.574), stearic acid (18:0, R_t_ = 21.315), oleic acid (18:1 cis, R_t_ = 21.799), and linoleic acid (18:2 cis, R_t_ = 22.615) were detected in all samples. The levels of fatty acids in sunflower oils showed no statistical significant differences for the samples obtained from irrigation lines 0, 100, and 500 as depicted in Table 4.

For the levels of palmitic acid (16:0) (ranging from 3.54 to 4.36% of total fatty acids), no statistically significant differences (*p*-value > 0.05) were observed between the levels of the acid (16:0) in 0 μg/L and all irrigation water lines according to ANOVA analysis. Similarly, for the levels of stearic acid (18:0) (ranging from 1.12 to 1.46% of total fatty acids), no statistically significant differences (*p*-value > 0.05) were observed between the levels of the acid (18:0) in 0 μg/L and all irrigation water lines according to ANOVA analysis.

For oleic acid (18:1 cis), the obtained levels ranged from 36.62 to 42.05% of total fatty acids and no statistically significant differences (*p*-value > 0.05) were observed between 0 μg/L and 100 μg/L and 0 μg/L and 500 μg/L, but statistically significant differences were observed between 0 μg/L and 1000 μg/L, 0 μg/L and 5000 μg/L, and 0 μg/L and 10,000 μg/L irrigation water lines. 

As for linoleic acid (18:2 cis), the obtained levels ranged from 52.46 to 58.00% of total fatty acids and no statistically significant difference (*p*-value > 0.05) were observed between 0 μg/L and 100 μg/L and 0 μg/L and 500 μg/L. However, statistically significant differences were observed between 0 μg/L and the higher irrigation water lines, i.e., between 0 μg/L and 1000 μg/L, 0 μg/L and 5000 μg/L, and finally between 0 μg/L and 10,000 μg/L.

## 4. Discussion

The simulation of the contaminated areas of Asopos and Messapia [8] in the greenhouse and the study of tubers, antioxidant enzymes, and carotenoids, which can be used as biomarkers of heavy metal pollution [19], has been a main focus of our research work over the past few years. In order to measure the absorption of chromium and nickel and to study the tolerance of sunflower (*Helianthus annuus*) to bioaccumulate and extract these toxic metals, we cultivated sunflowers, trying to give an alternative solution to the polluted area of Asopos and Messapia [1,4]. The purpose of this study, as already mentioned above, is to investigate the ability of the sunflower (*Helianthus annuus*) and specifically of the roots, shoots, leaves, and blossoms to bioaccumulate chromium and nickel and furthermore to assess the quantification of these two metals in the sunflower oil and the safety of the obtained oil for human consumption. 

The maximum absorption of chromium in shoots, leaves, and blossoms was mainly observed from the higher irrigation line (10,000 μg/L). It is important to note that according to our results, chromium stayed in the roots of the sunflower, while smaller concentrations were noticed in shoot, leaves, and blossoms by comparison [10]. Chromium is usually retained in vacuoles and cell walls of the root cells with low mobility within the plant. However, increased concentrations of Cr have been reported in the leaves of plants being grown in soils irrigated with different concentrations of Cr, but the transfer mechanism remains unknown [20]. After Cr is absorbed by roots from nutrient solution as Cr (III) or Cr (VI), it is poorly translocated elsewhere and largely retained in the root [10].

The highest absorption of nickel in the experiment for roots, shoots, and leaves of the sunflower was also observed from the last irrigation line (10,000 μg/L). The concentration of nickel absorbed by the roots was higher compared to that of shoots, leaves, and blossoms. Generally, the total amounts of chromium and nickel absorbed by the plant remained in the roots despite the fact that there were six different irrigation lines with different concentrations of Cr (VI) and Ni (II). In similar studies to ours, the higher absorbance of Ni was recorded in stems [21], whereas Ni in the leaves of sunflower have been observed to be selectively distributed to key points of the sheet (mesophyll) of the plant [17].

Sunflower’s capacity to extract heavy metals has been compared to Indian mustard and rapeseed, and it was found that sunflower can absorb Cd, Ni, Pb, and Zn more extensively. These results indicated that sunflower can grow on soil contaminated with Cr, Cu, and Zn, and it can also accumulate large amounts of Zn [22]. Sunflower is relatively resistant to high Zn soil concentrations; therefore, it can be used for Zn phytoextraction of moderately contaminated soil (approximately 200 mg Zn kg^−1^ soil) [23].

In the phytoextraction technology, the chosen plants should be able to absorb high amounts of heavy metals and translocate them from roots to shoots, leaves, and blossoms [13,14]. Many plants do not have the ability to achieve this transfer, for instance, plants with low biomass can hyper-accumulate metals in roots but they are not able to transfer them to other plant parts [24]. Roots were the main accumulation site for many heavy metals such as Pb. After four weeks, approximately 80–87% of the total Pb uptake was localized in plant roots with only 13–20% translocated to the aboveground plant parts. Their results indicated that sunflower, compared to other plant species (*N. tabacum* and *V. zizanioides*), best met prerequisites for a Pb-accumulating plant for use in remediating a Pb contaminated site [24]. Lead in the roots of sunflower reached 3.5 mg/kg and Zn reached 17.7 mg/kg. The accumulation of metals in the stems varied within a wide range and also depended on the type of crop and the element that was examined. Considerably lower values were ascertained in the leaves of sunflower. Lead in the leaves of sunflower reached 35.6 mg/kg. In general, the distribution of the heavy metals in the plant organs had a selective character [25]. The highest concentration of toxic metals was found in the roots of corn and sunflower (up to 1000 times higher than that of controls grown on non-contaminated soil). Relatively high concentrations of toxic metals were determined also in stems and leaves (10 times higher than in that of controls) [26]. Chromium, nickel, and lead showed similar trends. Respectively, the highest amounts were found in plant roots (for Cr 6.83 mg/kg, for Ni 5.04 mg/kg, and for Pb 7.76 mg/kg); sunflower has shown a higher ability to accumulate these elements than maize [27]. Cadmium accumulation in herbs was similar to those for Cr, Ni, and Pb, whereas a significantly higher amount of chromium was found in sunflower roots (7.02 mg/kg) [27]. In comparison to the other species, vetiver is the most tolerant to Pb, followed by castor bean, sunflower, and common buckwheat [28].

According to our statistical analysis, the differences between the irrigation lines with Cr (VI) and Ni (II) and the chromium and nickel levels in roots, shoots, leaves, and blossoms, in “two by two” comparison, were statistically significant, and there was also a positive strong correlation, which can be characterized as statistically significant, between them. 

Finally taking into account the sunflower oil results, it can be seen that the concentration of nickel was below the limit of detection and quantification. On the other hand, the concentrations of Cr were in the range of 0.029 to 0.071 μg/g in different irrigation water lines. Because of the fact that nickel levels in oils were below LOD or LOQ in all oil samples and due to the limited data obtained for Cr levels in oils, any statistical analysis for the levels of Cr and Ni in oils was not feasible. Our data strongly suggest, though, that the irrigation of plants with polluted water did not result in increased levels of either Ni or Cr in the obtained sunflower oil samples.

In previous research [29], the concentration of Cr was found to be below the limit of detection, and the concentrations of Ni and other selected metals were in a range of 0.1 to 1.5 μg/g and were comparable to other edible oils including sunflower oil; pumpkin seed oil samples contained higher amounts of Ni (6.1 μg/g). The levels of nickel in a comparison study were also not detected in sunflower oil and were lower than the LODs [30]. The presence of four elements, Cr, Cu, Fe, and Mn, was observed in all types of oils, and in the case of Cr, its concentration was higher in the vegetable oils other than olive oils. Nickel was also lower than the LODs [31]. 

The estimation of the risk to human health from the presence of chromium in food, particularly in vegetables, according to European Food Safety Authority (EFSA) is 0.3 mg/kg b.w. per day for Cr from the lowest Non-Observed Adverse Effect Level (NOAEL) identified in an (National Toxicology Program) NTP chronic oral toxicity study in rats [32]. The consumption of sunflower oil per day is approximately 20 mL so we can conclude that the oil, obtained in this study, is safe for consumption. Plant based edible oils, such as sunflower oil, are extensively used in daily life, and their quality has received great attention due to their influence on human nutrition and health. Those reasons led us to investigate further the lipid profile of the sunflower oil and, as mentioned above, considerable amounts of palmitic acid (16:0), stearic acid (18:0), oleic acid (18:1 cis), and linoleic acid (18:2 cis) were detected in all oil samples. The levels of palmitic (16:0) and stearic (18:0) acids exhibited no statistical significant differences between different irrigation water lines. The statistical analysis for oleic (18:1 cis) and linoleic (18:2 cis) acids showed a statistically significant increase and decrease respectively, between 0 μg/L and 1000 μg/L, 5000 μg/L, and 10,000 μg/L.

## 5. Conclusions

Given that both Cr and Ni tend to have an identical tendency to be phytoaccumulated, with the highest percentage concentrating in the roots [33], we have demonstrated here the ability of sunflower to phytoaccumulate significant amounts of these toxic metals. We have shown previously that the presence of Ni and Cr in Asopos River and Messapia areas causes cross contamination of the food chain [1,4]. The ability of the sunflower to bio remediate this water pollution is a promising technology which can give farmers an alternative agriculture species for farming, providing a final product (i.e., sunflower oil) that would not be cross-contaminated by harmful heavy metals. Also it is worth pointing out that due to the lipids profile and the results of chromium and nickel absorption, sunflower oil after a cleaning and removal process could be safe for consumption or for use as biofuel or other applications. 

According to EFSA recommendations on chromium intake (0.3 mg/kg b.w. per day) [32], and taking into consideration the daily consumption of sunflower oil (20 mL), our results validate the consumption of the oil as safe. Sunflower could also be used for fodder, technical purposes, and biodiesel whilst farmers would be able to decrease negative environmental consequences in the area.

## Figures and Tables

**Table 1:** Levels of Cr and Ni in plants by concentration in irrigation water.

	Cr (VI) and Ni (II) Concentration in Irrigation Water (μg/L)						
	0	100	500	1000	5000	10,000	
	Median ± SE	Median ± SE	Median ± SE	Median ± SE	Median ± SE	Median ± SE	rho ^a^ (*p*-value)
**Roots ^b^**							
*Levels of Cr (μg/g dry matter)*	0.1316 × 10^2^ ± 2.022	0.3766 × 10^2^ ± 11.59	3.041 × 10^2^ ± 39.63	5.577 × 10^2^ ± 122.7	17.99 × 10^2^ ± 547.0	6.708 × 10^2^ ± 412.4	0.945 (0.000)
*Levels of Ni (μg/g dry matter)*	0.1705 × 10^2^ ± 9.056	0.1369 × 10^2^ ± 3.220	0.5201 × 10^2^ ± 8.247	0.2335 × 10^2^ ± 16.01	0.5893 × 10^2^ ± 39.36	1.085 × 10^2^ ± 83.49	0.649 (0.001)
**Shoots ^c^**							
*Levels of Cr (μg/g dry matter)*	0.2250 ± 0.2204	0.1313 ± 0.1071	1.121 ± 0.4430	1.154 ± 0.9477	1.970 ± 1.392	4.000 ± 3.641	0.886 (0.000)
*Levels of Ni (μg/g dry matter)*	0.5715 ± 0.2535	0.4534 ± 0.4782	0.6328 ± 0.2579	0.2889 ± 0.1083	0.9707 ± 0.8005	4.954 ± 9.772	0.468 (0.004)
**Leaves ^d^**							
*Levels of Cr (μg/g dry matter)*	1.447 ± 0.5481	0.4819 ± 0.0956	2.218 ± 0.7129	4.057 ± 1.127	7.561 ± 1.116	0.1775 × 10^2^ ± 9.361	0.817 (0.000)
*Levels of Ni (μg/g dry matter)*	2.915 ± 0.9664	1.211 ± 0.4430	1.108 ± 2.379	2.292 ± 2.399	2.996 ± 2.913	0.1152 × 10^2^ ± 4.473	0.482 (0.003)
**Blossoms ^e^**							
*Levels of Cr (μg/g dry matter)*	1.551 ± 1.108	1.331 ± 1.952	3.144 ± 2.162	1.133 ± 0.6607	2.087 ± 0.7918	2.271 ± 1.700	0.268 (0.086)
*Levels of Ni (μg/g dry matter)*	5.236 ± 2.418	4.178 ± 0.7036	4.635 ± 1.795	2.047 ± 0.5885	7.966 ± 2.588	5.805 ± 3.523	0.293 (0.057)

^a^ Spearman’s rank correlation coefficient while *p*-value is given in brackets; ^b^ (*n* = 4 roots); ^c^ (*n* = 6 shoots); ^d^ (*n* = 6 leaves); ^e^ (*n* = 6 blossoms).

**Table 2:** Correlation Cr and Ni in shoots, leaves, blossoms, and roots by concentration in irrigation line.

	Cr (VI) and Ni (II) Concentration in Irrigation Water (μg/L)					
	0	100	500	1000	5000	10,000
	rho ^a^ (*p*-value)	rho ^a^ (*p*-value)	rho ^a^ (*p*-value)	rho ^a^ (*p*-value)	rho ^a^ (*p*-value)	rho ^a^ (*p*-value)
**Roots ^b^**						
*Levels of Cr/Ni*	−0.200 (0.800)	0.200 (0.800)	0.000 (1.000)	0.400 (0.600)	0.400 (0.600)	0.400 (0.600)
**Shoots ^c^**						
*Levels of Cr/Ni*	0.600 (0.208)	0.757 (0.084)	0.486 (0.329)	−0.143 (0.787)	−0.086 (0.872)	0.986 (0.000)
**Leaves ^d^**						
*Levels of Cr/Ni*	0.943 (0.005)	0.086 (0.872)	0.714 (0.111)	−0.086 (0.872)	−0.429 (0.397)	0.886 (0.019) ^
**Blossoms ^e^**						
*Levels of Cr/Ni*	0.543 (0.266)	−0.107 (0.819)	0.024 (0.955)	0.429 (0.397)	0.233 (0.546)	0.829 (0.042) ^

^a^ Spearman’s rank correlation coefficient while *p*-value is given in brackets; ^ Marginally significant difference (0.05 < *p* < 0.1) (Mann-Whitney non parametric test); ^b^ (*n* = 4 roots); ^c^ (*n* = 6 shoots); ^d^ (*n* = 6 leaves); ^e^ (*n* = 6 blossoms).

**Table 3:** Levels of nickel and chromium in sunflower oils.

Levels of Ni (II) and Cr (VI) in Irrigation Water (μg/L)	Ni (μg/g)	Cr (μg/g)
	Average ± SE	Average ± SE
**0**	<LOD ^a^	0.053 ± 0.02
**100**	<LOD ^a^	0.025 ± 0.01
**500**	<LOD ^a^	<LOQ ^b^
**1000**	<LOD ^a^	0.026 ± 0.01
**5000**	<LOD ^a^	0.026 ± 0.02
**10,000**	<LOD ^a^	0.028 ± 0.02
**Market Sample**	<LOD ^a^	<LOD ^b^

Average values ± standard error, for three oil samples per irrigation line (*n* = 3). ^a^ Limit of Detection = 0.078 (μg/g of sample), Limit of Quantification = 0.234 (μg/g of sample) for Nickel; ^b^ Limit of Detection = 0.008 (μg/g of sample), Limit of Quantification = 0.024 (μg/g of sample) for Chromium.

**Table 4:** Levels of fatty acids in sunflower oil.

Fatty Acids	Cr (VI) and Ni (II) Concentration in Irrigation Water (μg/L)					
	0	100	500	1000	5000	10,000
	Average ± SE	Average ± SE	Average ± SE	Average ± SE	Average ± SE	Average ± SE
**Palmitic acid (16:0)**	4.26 ± 0.09	3.89 ± 0.71 ^a^	4.05 ± 0.37 ^a^	3.54 ± 0.91 ^a^	3.56 ± 0.44 ^a^	4.36 ± 0.17 ^a^
**Stearic Acid (18:0)**	1.28 ± 0.87	1.46 ± 0.60 ^a^	1.33 ± 0.40 ^a^	1.38 ± 0.56 ^a^	1.12 ± 0.60 ^a^	1.13 ± 0.35 ^a^
**Oleic Acid (18:1 cis)**	36.95 ± 2.11	37.92 ± 3.51 ^a^	36.62 ± 2.34 ^a^	41.72 ± 0.86 ^b^	41.96 ± 3.94 ^b^	42.05 ± 2.46 ^b^
**Linoleic Acid (18:2 cis)**	57.82 ± 3.39	56.74 ± 2.72 ^a^	58.00 ± 2.12 ^a^	53.36 ± 0.64 ^b^	53.36 ± 4.93 ^b^	52.46 ± 2.29 ^b^

Average values ± standard error, for three oil samples per irrigation line (*n* = 3) (% total fatty acids). ^a^ Indicates no statistically significant differences (*p*-value > 0.05) between the 0μg/L concentration water line and all the other concentration water lines according to ANOVA analysis; ^b^ Indicates statistically significant differences (*p*-value < 0.05) between the 0μg/L concentration water line and the other concentration water lines (1000, 5000 and 10,000 μg/L) according to ANOVA analysis.

## Supplementary Materials

The following are available online at www.mdpi.com/2304-8158/6/7/51/s1, Figure S1: Levels of: (a) chromium and (b) nickel as determined by Atomic Absorption Spectroscopy (AAS) in roots, shoots, leaves, and blossoms in different irrigation lines. Table S1: Two by two comparisons of Cr and Ni between different concentrations in irrigation water.
