# Supplementary material for: Μetal Uptake by Sunflower (Helianthus annuus) Irrigated with Water Polluted with Chromium and Nickel
Source: Foods. 2017 Jul 17;6(7):51. doi: 10.3390/foods6070051 (PMC5532558; doi:10.3390/foods6070051)
Supplement: Supplementary file 1 [file foods-06-00051-s001.docx]

**Supplementary Figure 1** Levels of: (a) Chromium and (b) Nickel as determined by AAS in roots, shoots, leaves and blossoms in different irrigation lines.

**Supplementary Table 1** Two by Two Comparisons of Cr and Ni between Different Concentrations in Irrigation Water.

|  | **Cr** | **Ni** |  | **Cr** | **Ni** |  | **Cr** | **Ni** |  | **Cr** | **Ni** |
| --- | --- | --- | --- | --- | --- | --- | --- | --- | --- | --- | --- |
| **Roots** | **0** | **0** | **Shoots** | **0** | **0** | **Leaves** | **0** | **0** | **Blossoms** | **0** | **0** |
| 0 | 1 | 1 | 0 | 1 | 1 | 0 | 1 | 1 | 0 | 1 | 1 |
| 100 | 0.000*^#^ | 0.121 | 100 | 0.536 | 0.955 | 100 | 0.000*^#^ | 0.004*^#^ | 100 | 0.534 | 0.128 |
| 500 | 0.004*^#^ | 0.000*^#^ | 500 | 0.000*^#^ | 0.798 | 500 | 0.036^ | 0.059 | 500 | 0.228 | 0.613 |
| 1000 | 0.000*^#^ | 0.189 | 1000 | 0.003*^#^ | 0.029^ | 1000 | 0.001*^#^ | 0.573 | 1000 | 0.132 | 0.035^ |
| 5000 | 0.000*^#^ | 0.021^ | 5000 | 0.000*^#^ | 0.027^ | 5000 | 0.000*^#^ | 1.000 | 5000 | 0.328 | 0.012^ |
| 10000 | 0.093 | 0.002*^#^ | 10000 | 0.001*^#^ | 0.005*^#^ | 10000 | 0.029^ | 0.001*^#^ | 10000 | 0.065 | 0.445 |
| **Roots** | **100** | **100** | **Shoots** | **100** | **100** | **Leaves** | **100** | **100** | **Blossoms** | **100** | **100** |
| 100 | 1 | 1 | 100 | 1 | 1 | 100 | 1 | 1 | 100 | 1 | 1 |
| 500 | 0.005*^#^ | 0.000*^#^ | 500 | 0.000*^#^ | 0.779 | 500 | 0.000*^#^ | 0.918 | 500 | 0.240 | 0.132 |
| 1000 | 0.001*^#^ | 0.053 | 1000 | 0.001*^#^ | 0.101 | 1000 | 0.001*^#^ | 0.138 | 1000 | 0.699 | 0.002*^#^ |
| 5000 | 0.000*^#^ | 0.014^ | 5000 | 0.000*^#^ | 0.091 | 5000 | 0.000*^#^ | 0.002*^#^ | 5000 | 0.065 | 0.002*^#^ |
| 10000 | 0.106 | 0.003*^#^ | 10000 | 0.001*^#^ | 0.014^ | 10000 | 0.001*^#^ | 0.001*^#^ | 10000 | 0.041^ | 0.015^ |
| **Roots** | **500** | **500** | **Shoots** | **500** | **500** | **Leaves** | **500** | **500** | **Blossoms** | **500** | **500** |
| 500 | 1 | 1 | 500 | 1 | 1 | 500 | 1 | 1 | 500 | 1 | 1 |
| 1000 | 0.006*^#^ | 0.000*^#^ | 1000 | 0.864 | 0.130 | 1000 | 0.003*^#^ | 0.456 | 1000 | 0.065 | 0.002*^#^ |
| 5000 | 0.004*^#^ | 0.888 | 5000 | 0.000*^#^ | 0.021^ | 5000 | 0.000*^#^ | 0.063 | 5000 | 0.132 | 0.009*^#^ |
| 10000 | 0.190 | 0.004*^#^ | 10000 | 0.000*^#^ | 0.008*^#^ | 10000 | 0.036^ | 0.008*^#^ | 10000 | 0.394 | 0.240 |
| **Roots** | **1000** | **1000** | **Shoots** | **1000** | **1000** | **Leaves** | **1000** | **1000** | **Blossoms** | **1000** | **1000** |
| 1000 | 1 | 1 | 1000 | 1 | 1 | 1000 | 1 | 1 | 1000 | 1 | 1 |
| 5000 | 0.004*^#^ | 0.054 | 5000 | 0.088 | 0.012^ | 5000 | 0.000*^#^ | 0.456 | 5000 | 0.004*^#^ | 0.002*^#^ |
| 10000 | 0.432 | 0.003*^#^ | 10000 | 0.002*^#^ | 0.002*^#^ | 10000 | 0.065 | 0.009*^#^ | 10000 | 0.004*^#^ | 0.002*^#^ |
| **Roots** | **5000** | **5000** | **Shoots** | **5000** | **5000** | **Leaves** | **5000** | **5000** | **Blossoms** | **5000** | **5000** |
| 5000 | 1 | 1 | 5000 | 1 | 1 | 5000 | 1 | 1 | 5000 | 1 | 1 |
| 10000 | 0.045^ | 0.127 | 10000 | 0.008*^#^ | 0.224 | 10000 | 0.036^ | 0.003*^#^ | 10000 | 1.000 | 0.132 |

*Significant difference at level of significance (Bonferroni correction)

^#^ Significant difference at level of significance (Sidak correction)

^ Marginally significant difference (0.05<p<0.1) (Mann-Whitney non parametric test)
